# Synthesis and Antibacterial Activities of Amphiphilic Neomycin B-based Bilipid Conjugates and Fluorinated Neomycin B-based Lipids

**DOI:** 10.3390/molecules17089129

**Published:** 2012-08-02

**Authors:** Smritilekha Bera, Ramesh Dhondikubeer, Brandon Findlay, George G. Zhanel, Frank Schweizer

**Affiliations:** 1 Department of Chemistry, University of Manitoba, Winnipeg, MB R3T 2N2, Canada; 2 School of Chemical Sciences, Central University of Gujarat, Gandhinagar, Gujarat 382-030, India; 3 Department of Medical Microbiology, University of Manitoba, Winnipeg, MB R3E 3P4, Canada

**Keywords:** aminoglycosides, cationic amphiphiles, carbohydrates, antibacterials

## Abstract

Investigating the effect of lipid hydrophobicity on the activity of amphiphilic neomycin B conjugates, six polycationic amphiphiles (PAs) were created. Four of the new compounds incorporated either palmitic or arachidic di-lipid lysine tails, while two had single fluorinated undecanoic acid tails. The basicity of half of the compounds was increased through the incorporation of six guanidine moieties, in order to assess the effect of base strength on antimicrobial activity. A panel of ten bacteria was used for the testing, with seven strains obtained from the American Type Culture Collection series and three clinical isolates from Canadian Intensive Care Units. When compared to previous results with hydrocarbon monolipids the PAs all compounds were found to have reduced activity, though the hemolytic activity of the compounds with fluorinated tails was sharply reduced, with only a moderate reduction in antimicrobial activity.

## 1. Introduction

Much of our current antibiotic arsenal is derived from scaffolds developed forty or more years ago, allowing bacterial resistance to slowly accrue [1]. To combat tomorrow’s “superbugs” we will need new scaffolds [2], with antimicrobials designed specifically to combat the emergence of drug resistance. Polycationic lipids are one such scaffold, as agents such as chlorhexidine and benzalkonium chloride have been used as disinfectants for decades with little resistance development [3]. Unfortunately, these compounds are toxic to mammalian cells, limiting their use to topical applications [3,4]. Extensive research around analogues of cationic antimicrobial peptides such a magainin, mellitin and LL-37 has explored their potential to act as less toxic amphiphiles [4,5], but interest is beginning to move from the membrane-interacting behaviour of the peptides to their immunomodulatory properties and specific peptide-target interactions [6,7].

**Figure 1:**
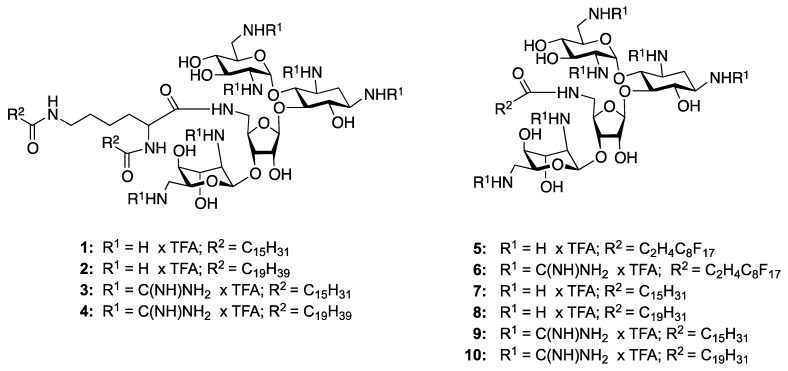
Structures of neomycin B-based bilipids **1**–**4**, the fluorinated monolipids **5** and **6**, and the previously reported hydrocarbon monolipid-neomycin-B amphiphiles **7**–**10** [9,12].

Starting from a class of compounds with known antibiotic action and limited ability to disrupt mammalian membranes, we have previously demonstrated the potential for a model aminoglycoside, neomycin, to act as a polycationic amphiphile (PA) [8,9,10,11,12]. In one study, coupling a single unit of palmitic acid to a C5"-amine restored good activity against a normally resistant strain of MRSA [12]. Reducing the size of the lipid tail to twelve carbons eliminated activity, confirming that the gain was due to an increase in aminoglycoside hydrophobicity. In this work we attempted to increase PA self-association in both the bacterial membrane and solution, through the incorporation of either a fluorocarbon tails or two hydrophobic tails (Figure 1). Self-association in the membrane was postulated to decrease the concentration required for membrane disruption, by forming pockets of high PA concentration, while self-association in solution could lead to micelle formation and general exclusion of the hydrophobic portion of the amphiphiles from circulation.

## 2. Results and Discussion

**Scheme 1:**
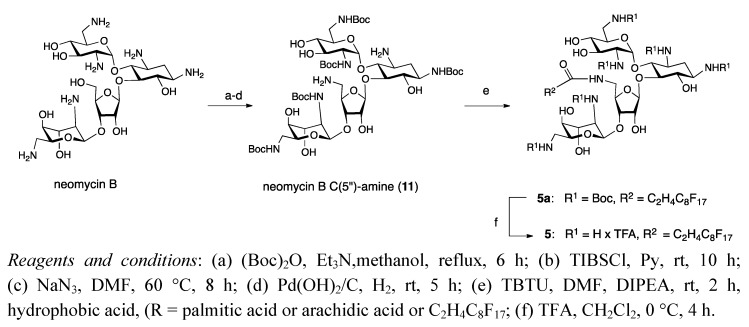
Strategy for the synthesis of neomycin B C(5")-modified analog **5**.

While an appealing scaffold for PA design, the polyfunctional nature of neomycin necessitates multiple protection/deprotection steps for selective modifications. This is often perceived as a serious limitation to drug development, but we have developed an efficient series of reactions for modification of the 5"-OH moiety. The four step synthesis begins with protection of the amine functionalities, followed by conversion of the 5"-OH to a primary amine for standard amide bond formation (Scheme 1). Crystal structures have confirmed that this site is not required for tight binding to the ribosome [13], and in the past this set of reactions was used to create a number of monolipid analogues [8,12], including some with the standard amine groups converted into more basic guanidines [9].

**Scheme 2:**
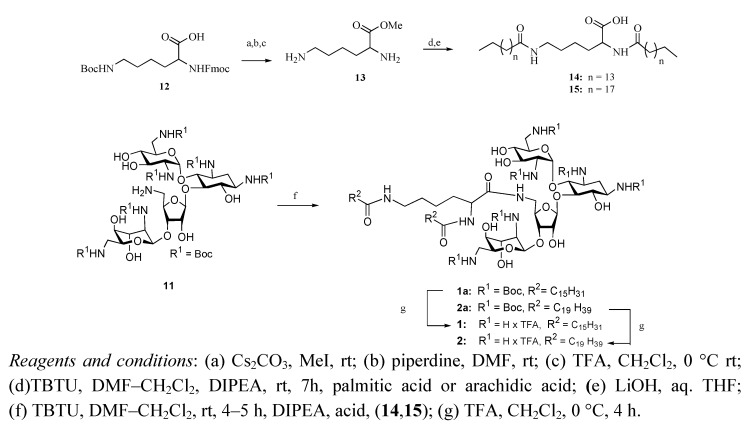
Synthesis of neomycin B C(5")-modified bilipids **1** and **2**.

Incorporation of two hydrophobic tails required the use of a specially modified carboxylic acid. Due to its ready availability we began with the protected amino acid Fmoc-Lys(Boc)-OH (Scheme 2), first converting the carboxylic acid to a methyl ester via cesium carbonate and methyl iodide. 

The amino acid protecting groups were then sequentially cleaved under standard conditions and either palmitic or arachidic acid was coupled to both free amines, with TBTU as the coupling agent. A mixture of DMF and DCM was required to effect solubilization during this reaction, due to the highly hydrophobic nature of arachidic acid. Following the coupling the methyl ester was cleaved by lithium hydroxide in aqueous THF to furnish dilipids **14** and **15**.

Interested in the effect of guanidinylation on PA activity, two different types of lipid conjugated neomycin B amphiphiles were produced. Synthesis of both types began with protection of the amine moieties with Boc anhydride in a refluxing mixture of TEA and MeOH. The primary hydroxyl group was then activated as a triisopropylbenzenesulfonate ester, and the sulfonate was then displaced *via* S_N_2 nucleophilic attack by sodium azide. The bulky sulfonyl chloride was chosen for its preference for unhindered primary alcohols, which reduced substitution at the six secondary alcohols [14].

Reduction of the azide was accomplished via catalytic hydration by palladium hydroxide on charcoal and hydrogen gas, affording the free 5"-amino hexa-Boc protected neomycin B, **11**. This compound was then coupled to dilipids **14** and **15**, and following purification by column chromatography the protecting groups were cleaved with 95:5 TFA: H_2_O. Trituration in 98:2 Et_2_O/MeOH removed any residual hydrophobic impurities, providing dilipid neomycin salts **1** and **2** in good yield.

Compound **11** was also used to produce a monolipid analogue with a fluorinated tail, compound **5**, again via standard peptide coupling reagents. A fluorinated undecanoic acid analogue was selected, due to a mix of commercial availability and stability of the final amide. Previous work with perfluorinated acids has shown that the resulting amide bond is not stable to hydrolysis, due to the strong acidity of the conjugate ester [15]. The addition of two methylene spacers between the fluorocarbon region and the carboxylic acid eliminated this hydrolysis, but limited commercial availability to tails that were a total of eleven carbons long or shorter. As this work was intended to explore the effect of self-associating tails on PA activity the longest commercially available lipid was selected. While significantly shorter the fluorinated undecanoic acid tail is nearly twice the molecular weight of palmitic acid, and so compounds **1** and **5** were similar in final molecular weight.

**Scheme 3:**
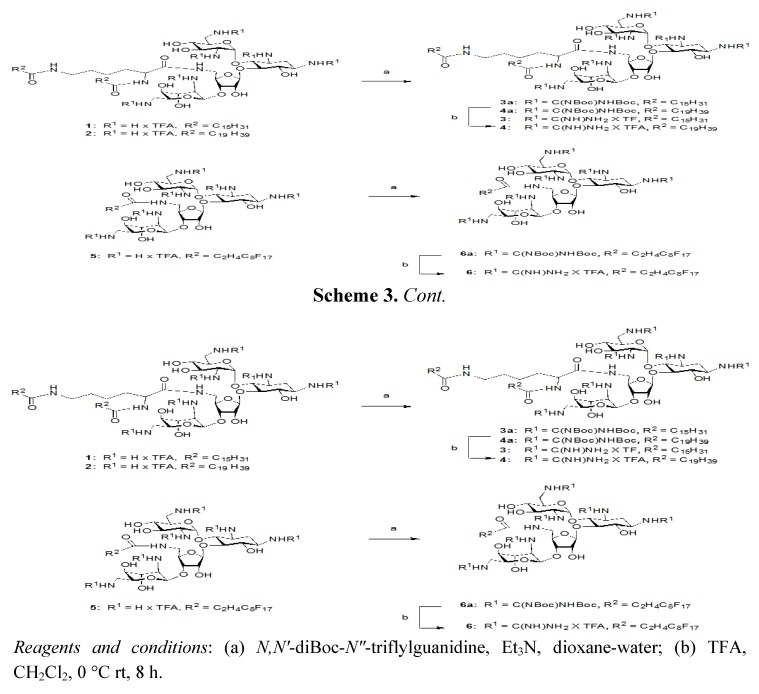
Synthesis of neomycin B C(5")-modified guanidinylated analogs **3**, **4** and **6**.

The second set of PAs was constructed by replacing the amines of neomycin B with guanidine groups, through the use of the commercially available *N*,*N'*-Diboc-*N"*-triflylguanidine [16] (Scheme 3). 

PAs **1**–**3** were reacted over a period of three to four days, followed by extraction and column chromatography to give compounds **3a**, **4a**, and **6a**. Treatment with 1:1 TFA: DCM for four h cleaved the Boc protecting groups to give compounds **3**, **4**, and **6**; which were purified via trituration in 98:2 Et_2_O : MeOH.

Polycationic amphiphiles in hand, we then determined their antimicrobial activity against a panel of both American Type Culture Collection (ATCC) and clinically relevant bacterial strains. Pathogenic isolates were obtained during the CAN-ICU surveillance study [17], and represent “clinically significant” strains of *Escherichia coli* and *Pseudomonas aeruginosa*. ATCC strains used in this testing allow for comparison to previous PA studies [18], with gentamicin and neomycin serving as positive controls.

We were especially interested in comparing the activity of our dilipid and fluorinated lipid aminoglycosides to the activity of PAs **7**–**10** (Figure 1), which have been reported previously [9,12]. In line with previous results involving monolipid tails [9,12], PAs **1**–**6** demonstrated higher activity against Gram positive bacteria than Gram negative (Table 1), with the palmitoyl guanidylated dilipid **3** being the most active compound overall. Guanidinylation increased the activity of the amphiphiles against Gram positive bacteria 2–8 fold, with both strains of *Staphylococcus epidermidis* highly susceptible, in line with the activity of neomycin against these strains. The greatest improvement in activity was against the Gram negative bacteria *P. aeruginosa*, where the MIC decreased from 512 ug/mL for neomycin to 32 ug and 16 ug for compound **3** (against ATCC strain 27853 and CAN-ICU strain 62308, respectively). While compound **3** was more active than the PAs with fluorinated monolipids, both **5** and **6** had improved activity over the remaining dilipids, perhaps due to the potential for the dilipids to form either insoluble aggregates.

**Table 1:** Antibacterial and hemolytic activity (MIC) in µg/mL of various neomycin B-based mono- and bilipids **1**–**6**.

Control Organism	Genta-mycin	Neo- mycin	1	2	3	4	5	6
*S. aureus* ATCC29213	1	1	16	32	4	64	16	8
MRSA ATCC33592	2	256	16	128	4	128	64	8
*S. epidermidis* ATCC14990	0.25	0.25	4	16	2	16	8	8
MRSE CAN-ICU 61589	32	0.5	4	16	4	64	4	4
*E. faecalis* ATCC29212	n.d.	16	16	128	8	128	64	16
*E. facium* ATCC27270	n.d.	4	16	128	4	128	32	2
*S. pneumoniae* ATCC49619	4	32	128	128	64	>128	64	32
*E. coli* ATCC25922	1	4	32	32	64	>128	32	16
*E. coli* CAN-ICU 61714	128	8	32	64	32	>128	32	32
*E. coli* CAN-ICU 63074	8	n.d.	64	64	32	>128	64	64
*P. aeruginosa* ATCC27853	8	512	128	>256	32	128	128	64
*P. aeruginosa* CAN-ICU 62308	128	512	64	128	16	128	32	8
% hemolysis at 100 µg/mL/ (500 µg/mL)	<0.4 (<0.4)	<0.4 (<0.4)	n.d. (n.d.)	n.d. (n.d.)	n.d. (n.d.)	n.d. (n.d.)	1.1 (9.4)	1.8 (11.9)

Moving from a monolipid to a dilipid scaffold appeared to reduce the activity in the context of palmitic and arachidic tails. In general there was a 2–4 fold reduction in activity against most bacterial species, with no significant difference in reduction between Gram positive and Gram negative strains. PAs with fluorinated lipid tails were less active than the palmitoyl monolipids, which had a similar mass, but demonstrated significantly reduced toxicity towards eukaryotic cells. While hemolysis data was not obtained for PAs **1**–**4**, we are encouraged by this broadening of the therapeutic window.

## 3. Experimental

### 3.1. General Methods

NMR spectra were recorded on a Bruker Avance 300 spectrometer (300 MHz for ^1^H-NMR, 75 MHz for ^13^C) or AMX 500 spectrometer (500 MHz for ^1^H-NMR). Optical rotation was measured at a concentration of g/100 mL, with a Perkin-Elmer polarimeter (accuracy 0.002°). GC-MS analyses were performed on a Perkin-Elmer Turbomass-Autosystem XL. Analytical thin-layer chromatography was performed on precoated silica gel plates, with spot visualization via ultraviolet light and/or by staining with ninhydrin solution in ethanol. Chromatographic separations were performed on a silica gel column by flash chromatography (Kiesel gel 40, 0.040–0.063 mm; Merck). Yields are given after purification, unless explicitly stated. Reactions requiring anhydrous conditions were performed under nitrogen or argon gas.

### 3.2. Synthesis of Lysine-Dilipid Conjugates ***14*** and ***15***

Commercially available side chain protected Fmoc-Lys(NHBoc)-COOH was converted to methyl ester using TBTU in DMF followed by removal of Fmoc- and Boc-protecting groups followed by condensation to palmitic acid/arachidic. Ester hydrolysis was performed with LiOH in a mixture of THF/H_2_O (2:1).

*N,N-di-Hexadecanoyl-Lys-acid* (**14**). Yield = 65%; ^1^H-NMR (300 MHz, CD_3_OD and CDCl_3_): δ 6.22 (d, 1H, *J* = 7.9 Hz), 5.76 (t, 1H, *J* = 5.0 Hz), 4.55 (m, 1H), 3.22 (t, 2H, *J* = 6.3 Hz), 2.19 (m, 4H), 1.84 (m, 2H), 1.61 (m, 4H), 1.41 (m, 4H), 1.24 (s, 48H), 0.87 (t, 6H, *J* = 6.6 Hz); EIMS: calcd for C_38_H_74_NaN_2_O_4_^+^ 645.56 Found: 645.88 [M+Na]^+^.

*N,N-di-Nonadecanoyl-Lys-acid* (**15**). Yield = 61%; ^1^H-NMR (300 MHz, CD_3_OD and CDCl_3_): δ 6.22 (d, 1H, *J* = 7.9 Hz), 5.76 (t, 1H, *J* = 5.0 Hz), 4.55 (m, 1H), 3.32 (t, 2H, *J* = 6.3 Hz), 2.19 (m, 4H), 1.84 (m, 2H), 1.73–1.41 (m, 8H), 1.24 (s, 64H), 0.87 (t, 6H, *J* = 6.6 Hz); EIMS: calcd for C_46_H_90_N_2_NaO_4_^+^ 775.69 Found: 775.97 [M+Na]^+^.

### 3.3. General Procedure for Coupling Reaction of Neomycin B-based Amine ***11***

To a solution of neomycin amine **11** (1 equiv.) [12] in dry DMF, TBTU (2 equiv.), the lipid of choice (1 equiv.) and DIPEA (3 equiv.) were added. The mixture was stirred at room temperature for 2 h, then extracted with water and ethyl acetate. The ethyl acetate layer was washed with water, brine, dried over sodium sulfate and concentrated. The crude residue was purified using flash silica gel (CH_2_Cl_2_/MeOH). Spectroscopic data is provided below.

### 3.4. General Procedure for Final Deprotection

All Boc-protected lipids were treated with 95:5 TFA–H_2_O at 0 °C for 3 min, following which the solvent was removed at reduced pressure. To the residue 2% methanol in ether was added and the solvent was decanted to get the solid neomycin-lipid conjugate as salt. The spectroscopic data is given below.

*5"-N-(N,N-di-Hexadecanoyl-Lys)-1,3,2',6',2''',6'''-hexa-N-(tert-butoxycarbonyl)-5''-deoxy-neomycin* (**1a**). Yield = 78%; *R*_f_ 0.35 (MeOH/CH_2_Cl_2_ 1:9); ^1^H-NMR (300 MHz, CD_3_OD): δ 5.48 (br s, 1H), 5.09 (br s, 1H), 4.88 (s, 1H), 4.31 (d, 1H, *J* = 3.4 Hz), 4.07 (t, 1H, *J* = 4.4 Hz), 3.96–3.80 (m, 5H), 3.76 (m, 2H), 3.56 (m, 4H), 3.48 (br t, 3H, *J* = 3.9 Hz), 3.39 (m, 4H), 3.27 (d, 2H, *J* = 7.6 Hz), 3.20 (m, 2H), 3.12 (m, 1H), 2.34 (m, 3H), 1.97 (dt, 1H, *J* = 4.3, 11.1 Hz), 1.64 (m, 4H), 1.51–144 (m, 56H), 1.43 (s, 9H), 1.29 (s, 45H), 0.90 (t, 6H, *J* = 6.1 Hz); ^13^C-NMR (75 MHz, CD_3_OD): δ 176.6 (amide C=O), 176.4 (×2) (peptide amide C=O), 158.9–158.7 (Boc-CO), 112.1, 100.4, 98.8 (anomeric carbons), 88.4, 80.8–80.3, 79.4, 76.2, 75.6, 74.5, 74.1, 73.4, 71.7, 69.0, 69.3, 56.8, 53.6, 51.3, 52.5, 43.9, 42.7, 42.5, 38.9, 37.0–23.7 (aliphatic CH_2_), 14.5 (aliphatic CH_3_); [α]_D_^25^ = +32.0 (c 0.53, MeOH); EIMS: calcd for C_91_H_167_N_9_NaO_27_^+^ 1841.22 Found: 1841.45 [M+Na]^+^; Anal. Calcd. for C_91_H_167_N_9_O_27_ C, 60.08; H, 9.25; N, 6.93; Found: C, 60.33; H, 9.45; N, 7.03.

*1,3,2',6',2''',6'''-Hexaammonium-5"-deoxy-5"-N-(N,N-di-hexadecanoyl-Lys)-neomycin hexakis-(trifluoroacetate)* (**1**). Yield = 89%; *R*_f_ 0.10 (NH_4_OH/MeOH/CH_2_Cl_2_ 2:9:4); ^1^H-NMR (300 MHz, CD_3_OD): δ 5.86 (d, 1H, *J* = 3.1 Hz), 5.40 (d, 1H, *J* = 4.4 Hz), 5.30 (s, 1H), 4.41 (t, 1H, *J* = 5.4 Hz), 4.30 (m, 2H), 4.22 (m, 1H), 4.16 (m, 2H), 4.08 (t, 2H, *J* = 5.9 Hz), 3.99 (t, 2H, *J* = 3.6 Hz), 3.89 (t, 2H, *J* = 8.8 Hz), 3.68 (m, 4H), 3.52 (dd, 2H, *J* = 2.6, 11.1 Hz), 3.45 (m, 3H), 3.41 (t, 1H, *J* = 3.6 Hz), 3.37 (d, 1H, *J* = 1.6 Hz), 3.24 (m, 1H), 3.20 (m, 1H), 2.48 (d, 2H, *J* = 11.8 Hz), 2.26 (d, 3H, *J* = 7.8 Hz), 2.08 (d, 2H, *J* = 11.8 Hz), 1.61 (m, 3H), 1.29 (s, 52H), 0.90 (t, 6H, *J* = 6.1 Hz); ^13^C-NMR (75 MHz, CD_3_OD): δ 177.4 (amide C=O), 177.3 (×2) (peptide amide C=O), 164.0–162.7 (TFA, q with *J ^2^_CF_* ~ 34.8 Hz), 124.1–112.4 (q with *J ^1^_CF_* ~ 292.0 Hz), 109.5, 97.2, 96.7 (anomeric carbons), 86.6, 83.4, 78.1, 76.6, 76.4, 75.6, 74.1, 74.0, 72.9, 72.5, 72.0, 71.9, 71.8, 69.4, 67.6, 64.9, 55.4, 52.9, 51.1, 50.3, 41.9, 41.7, 37.2–23.7 (aliphatic CH_2_), 14.7 (CH_3_); [α]_D_^25^ = +24.0 (c 1.58, MeOH); EIMS: calcd for C_61_H_120_N_9_O_15_^+^1218.88 Found: 1219.09 [M+H]^+^; Anal. Calcd. for C_73_H_125_F_18_N_9_O_27_ C, 46.08; H, 6.62; F, 17.97; N, 6.63; Found: C, 46.23; H, 6.76; F, 18.01; N, 6.46.

*5"N-(N,N-di-Nonadecane-Lys)-1,3,2',6',2''',6'''-hexa-N-(tert-butoxycarbonyl)-5"-deoxy-neomycin* (**2a**). Yield = 70%; *R*_f_ 0.33 (MeOH/CH_2_Cl_2_ 1:9); ^1^H-NMR (300 MHz, 2:1 mixture of CD_3_OD and CDCl_3_): δ 5.08 (m, 2H), 4.74 (m, 1H), 4.36 (m, 2H), 4.22 (m, 2H), 4.03 (t, 1H, *J* = 7.3 Hz), 3.99 (m, 4H), 3.79 (m, 2H), 3.70 (m, 2H), 3.55 (m, 3H), 3.46 (m, 4H), 3.43 (m, 2H), 3.25 (m, 2H), 3.13 (m, 1H), 2.34 (m, 2H), 2.24 (t, 2H, *J* = 7.9 Hz), 1.99 (m, 2H), 1.69 (m, 2H), 1.55–1.40 (m, 72H), 1.28 (s, 54H), 0.89 (t, 6H, *J* = 7.1 Hz); ^13^C-NMR (75 MHz, CD_3_OD): δ 176.2 (amide C=O), 175.7 (×2) (peptide amide C=O), 158.3–157.2 (Boc-CO), 101.6, 100.3, 97.6 (anomeric carbons), 87.5, 81.4–80.6, 78.4, 76.4, 75.5, 75.3, 74.5, 73.5, 73.3, 72.4, 71.4, 68.9, 57.1, 56.1, 53.4, 52.3, 44.2, 42.8, 40.0, 38.0, 37.7–24.0 (aliphatic CH_2_), 14.4(CH_3_); [α]_D_^25^ = +55.0 (c 0.73, MeOH); EIMS: C_99_H_183_N_9_NaO_27_^+^ 1953.32 Found: 1953.67 [M+Na]^+^; Anal. Calcd. for C_99_H_183_N_9_O_27_ C, 61.56; H, 9.55; N, 6.53; Found: C, 62.22; H, 10.09; N, 6.53.

*1,3,2',6',2"',6"'-Hexaammonium-5"-deoxy-5"-N-(N,N-di-nonadecanoyl-Lys)-neomycin hexakis-(trifluoroacetate)* (**2**). Yield = 87%; *R*_f_ 0.10 (NH_4_OH/MeOH/CH_2_Cl_2_ 2:9:4); ^1^H-NMR (300 MHz, 2:1 mixture of CD_3_OD and CDCl_3_): δ 5.40 (d, 1H, *J* = 3.1 Hz), 5.25 (d, 1H, *J* = 4.4 Hz), 5.20 (s, 1H), 4.79 (m, 1H), 4.62 (m, 1H), 4.40–4.00 (m, 9H), 3.89 (t, 1H, *J* = 5.9 Hz), 3.68 (m, 2H), 3.60 (m, 1H), 3.44 (m, 6H), 3.22 (m, 4H), 2.45 (m, 1H), 2.21 (d, 2H, *J* = 8.1 Hz), 2.19 (d, 2H, *J* = 7.1 Hz), 2.00 (d, 2H, *J* = 6.4 Hz), 1.60 (m, 5H), 1.22 (s, 70H), 0.90 (t, 6H, *J* = 7.2 Hz); ^13^C-NMR (75 MHz, CD_3_OD): δ 177.2 (amide C=O), 176.1 (×2) (peptide amide C=O), 164.0–162.7 (TFA, q with *J ^2^_CF_* ~ 34.8 Hz), 124.1–112.4 (q with *J ^1^_CF_* ~ 292.0 Hz), 109.5, 97.2, 96.3 (anomeric carbons), 86.3, 83.0, 79.2, 78.2, 75.9, 75.5, 73.8, 72.9, 72.4, 72.1, 71.9, 71.7, 69.4, 69.0, 61.9, 55.9, 52.9, 51.8, 50.3, 41.9, 41.7, 40.6, 40.5, 37.5–23.7 (aliphatic CH_2_) 14.7 (CH_3_); [α]_D_^25^ = +55.0 (c 0.71, MeOH); EIMS: calcd for C_69_H_136_N_9_O_15_^+^ 1331.01 Found: 1331.45 [M+H]^+^; Anal. Calcd for C_81_H_141_F_18_N_9_O_27_ C, 48.28; H, 7.05; F, 16.97; N, 6.26 Found: C, 48.28; H, 7.05; F, 16.97; N, 6.26.

*5"-N-(Heptadecafluoro-decanoyl)-1,3,2',6',2"',6"'-hexa-N-(tert-butoxycarbonyl)-5"-deoxy-neomycin* (**5a**). Yield = 88%; R_f_ 0.56 (MeOH/CH_2_Cl_2_ 1:9); ^1^H-NMR (300 MHz, CD_3_OD): δ 5.54 (br s, 1H), 5.10 (br s, 1H), 4.89 (br s, 1H), 4.34 (d, *J* = 4.38, 1H), 4.11–4.04 (m, 2H), 3.97–3.85 (m, 4H), 3.79–3.72 (m, 2H), 3.59–3.47 (m, 6H), 3.44–3.36 (m, 3H), 3.28–3.18 (m, 2H), 3.08 (dd, *J* = 8.6, 13.6 Hz, 1H), 2.82–2.47 (m, 4H), 1.97–192 (d, *J* =12.6 Hz, 1H), 1.67–1.60 (m, 2H), 1.51–1.43 (m, 54H), 1.39–1.22 (m, 2H); ^13^C-NMR (75 MHz, CD_3_OD): δ 173.0 (amide-CO) 159.1, 158.9, 158.7, 158.6, 158.3, 157.9 (Boc-CO), 112.3, 100.3, 98.7 (anomeric carbons), 88.6, 81.0, 80.8, 80.7, 80.4, 80.3, 76.3, 75.5, 74.5, 73.4, 72.7, 72.5, 71.7, 69.1, 56.8, 53.6, 52.7, 51.3, 44.2, 42.7, 42.0, 35.7, 28.9–27.5 (Boc CH_3_ and aliphatic CH_2_); EIMS: calcd. for C_64_H_99_F_17_N_7_O_25_+ 1688.64. Found: 1688.45 [M+H]^+^.

*1,3,2',6',2''',6"'-Hexaammonium-5"-deoxy-5"-N-(heptadecafluoro-decanoyl)-neomycin hexakis-(trifluoroacetate)* (**5**). Yield = 89%; R_f_ 0.14 (NH_4_OH/MeOH/CH_2_Cl_2_ 1:5:5); ^1^H-NMR (300 MHz, CD_3_OD): δ 5.85 (d, *J* = 3.7 Hz, 1H), 5.40 (d, *J* = 4.8 Hz, 1H), 5.29 (br s, 1H), 4.41–4.33 (m, 1H), 4.25 (m, 4H), 4.14 (m, 1H), 4.08 (m, 1H), 4.00 (m, 2H), 3.88 (dd, 1H, *J* = 9.0, 7.8 Hz), 3.73–3.66 (m, 2H), 3.64 (m, 1H), 3.55–3.34 (m, 8H), 3.26–3.21 (m, 1H), 3.16 (dd, 1H, *J* = 13.4, 8.1 Hz), 2.58 (m, 3H), 2.46 (m, 1H), 2.07 (m, 1H); ^13^C-NMR (75 MHz, CD_3_OD): δ 173.70 (NH-CO-), 163.3 (TFA), 123.93, 120.38, 120.09, 120.07, 119.86, 119.82 (F attached), 117.2 (TFA), 109.61, 97.33, 96.71 (anomeric carbons), 86.55, 83.19, 78.18, 76.50, 75.58, 73.99, 73.05, 72.04, 71.94, 69.48, 69.33, 69.18, 66.87, 55.03, 52.88, 51.14, 50.31, 42.21, 41.96, 41.68, 29.43, 27.51; EIMS: calcd. for C_34_H_51_F_17_N_7_O_13_+ 1088.32. Found: 1088.45 [M+H]^+^.

### 3.5. General Procedure for the Guanidinylation of Aminoglycosides

*N*,*N*'-DiBoc-*N*"-triflylguanidine was purchased from Fluka and used without further purification. To a solution of aminoglycoside (5 amines, 0.054 mmol) in H_2_O (0.5 mL) was added 1,4-dioxane (2.5 mL) and *N*,*N*'-diBoc-*N*"-triflylguanidine (0.82 mmol) in alternating portions so the solution remained relatively clear. After 5 min, NEt_3_ (0.82 mmol) was added at room temperature. After 3–4 days, the 1,4-dioxane was removed under reduced pressure. The remaining residue and H_2_O was extracted with CH_2_Cl_2_ (3 × 10 mL), washed with H_2_O and brine, and dried (MgSO_4_). The fully guanidinylated product can be isolated by flash column chromatography (FCC) on silica gel (CH_2_Cl_2_/MeOH).

### 3.6. General Procedure for the Deprotection of Guanidinoglycosides

A solution of TFA: CH_2_Cl_2_ (1:1, 1 mL) was added to the protected guanidinoglycoside (0.041 mmol) at room temperature. After approximately 4 h, the solution was diluted with toluene, concentrated in vacuo, and dissolved in H_2_O. Subsequent lyophilization of H_2_O provided the deprotected guanidinoglycoside as a fluffy white powder.

*5"-Deoxy-5"-N-(N,N-di-hexadecanoyl-Lys)-1,3,2',6',2"',6'''-hexa-N-(tert-butoxycarbonyl-guanidino)-5"-deoxy-neomycin* (**3a**). Yield = 75%; *R*_f_ 0.33 (MeOH/CH_2_Cl_2_ 1:9); ^1^H-NMR (300 MHz, CD_3_OD): δ 5.62 (s, 1H), 5.22 (s, 2H), 5.08 (s, 1H), 4.55 (m, 1H), 4.38 (m, 2H), 4.23 (m, 3H), 4.10 (m, 4H), 3.99 (m, 2H), 3.91–3.77 (m, 2H), 3.72 (m, 2H), 3.63–3.50 (m, 6H), 3.40 (t, 1H, *J* = 5.3 Hz), 3.20 (m, 1H), 2.26 (m, 4H), 1.72 (m, 2H), 1.50 (s, 128H), 1.30 (s, 36H), 0.89 (t, 6H, *J* = 7.1 Hz); ^13^C-NMR (75 MHz, CD_3_OD, HSQC): δ 176.5 (amide C=O), 176.2 (×2) (peptide amide C=O), 164.4–164.1 (Boc C=O), 158.0–157.4 (guanidine C=NH), 154.4–153.7 (Boc C=O), 111.3, 97.7, 95.8 (anomeric carbons), 88.3, 84.8–84.1, 82.6, 80.7–80.4, 79.6, 77.3, 76.5, 75.3, 74.2, 73.1, 72.6, 71.7, 71.2, 68.4, 56.4, 54.2, 52.9, 50.8, 51.7, 44.4, 43.6, 43.3, 41.8, 39.7–22.6 (aliphatic CH_3_) 13.4 (CH_3_); [α]_D_^25^ = +56.0 (c 0.56, MeOH); EIMS: calcd for C_127_H_227_N_21_NaO_39_^+^ 2693.64 Found: 2693.89 [M+Na]^+^; Anal. Calcd. for C_127_H_227_N_21_O_39_ C, 57.08; H, 8.56; N, 11.01; Found: C, 57.67; H, 9.11; N, 11.41.

*1,3,2',6',2''',6'''-Hexaammonium-guanidinyl-5"-deoxy-5"-N-(N,N-di-hexadecanoyl-Lys)-neomycin hexakis(trifluoroacetate)* (**3**). Yield = 81%; *R*_f_ 0.10 (NH_4_OH/MeOH/CH_2_Cl_2_ 2:9:4); ^1^H-NMR (300 MHz, CD_3_OD): δ 5.64 (d, 1H, *J* = 2.7 Hz), 5.18 (m, 2H), 5.06 (m, 1H), 5.03 (d, 1H, *J* = 5.3 Hz), 4.77 (m, 1H), 4.23 (m, 1H), 4.15–4.08 (m, 3H), 4.01 (m, 2H), 3.80–3.77 (m, 3H), 3.67 (m, 3H), 3.57–3.48 (m, 6H), 3.42 (m, 2H), 3.37 (m, 1H), 3.19 (t, 1 H, *J* = 7.3 Hz), 2.24 (m, 3H), 2.19 (m, 2H), 1.72 (m, 2H), 1.61 (m, 4H), 1.46 (m, 2H), 1.29 (s, 49H), 0.91 (t, 6H, *J* = 6.5 Hz); ^13^C-NMR (75 MHz, CD_3_OD): δ 177.6 (amide C=O), 177.4 (×2) (peptide amide C=O), 159.6, 159.2 (×4), 158.6 (guanidine C=NH), 100.6, 97.9, 97.6 (anomeric carbons), 88.4, 86.6, 83.9, 82.4, 79.6, 79.4, 76.4, 75.2, 74.0, 72.7, 72.0, 71.4, 70.9, 69.8, 57.1, 55.4, 52.9, 51.7, 50.3, 43.6, 41.9, 41.7, 38.9–23.7 (aliphatic CH_2_), 14.7 (aliphatic CH_3_); [α]_D_^25^ = +44.0 (c 0.67, MeOH); EIMS: calcd for C_67_H_131_N_21_O_15_^+^ 1471.01 Found: 1471.22 [M+H]^+^; Anal. Calcd. for C_79_H_137_F_18_N_21_O_27_ C, 44.03; H, 6.41; F, 15.87; N, 13.65; Found: C, 44.22; H, 6.82; F, 16.11; N, 14.15.

*5''-Deoxy-5"-N-((N,N-di-nonadecanoyl-Lys)-1,3,2',6',2''',6'''-hexa-N-(tert-butoxycarbonylguanidino)-5"-deoxyneomycin* (**4a**). Yield = 71%; *R*_f_ 0.32 (MeOH/CH_2_Cl_2_ 1:9); ^1^H-NMR (300 MHz, CD_3_OD and CDCl_3_): δ 5.74 (s, 1H), 5.05 (m, 2H), 4.74 (m, 1H), 4.55 (m, 1H), 4.38 (m, 2H), 4.18 (m, 3H), 4.03 (m, 1H), 3.99 (m, 2H), 3.83 (m, 3H), 3.72–3.35 (m, 6H), 3.23 (t, 2H, *J* = 9.3 Hz), 3.17 (d, 1H, *J* = 6.7 Hz), 3.43 (d, 3H, *J* = 13.4 Hz), 2.25 (t, 2H, *J* = 6.9 Hz), 2.17 (t, 2H, *J* = 7.3 Hz), 2.03 (m, 2H), 1.72 (m, 2H), 1.50 (s, 108H), 1.30 (s, 74H), 0.89 (t, 6H, *J* = 7.1 Hz); ^13^C-NMR (75 MHz, CD_3_OD, HSQC): δ 176.2 (amide C=O), 176.1 (×2) (peptide amide C=O), 164.6–163.9 (Boc C=O), 158.2–157.3 (guanidine C=NH), 154.4–153.7 (Boc C=O), 111.3, 97.7, 95.8 (anomeric carbons), 86.8, 85.1–84.0, 82.0, 80.9–80.3, 78.7, 78.5, 75.3, 74.2, 73.5, 72.6, 71.7, 71.2, 70.6, 69.4, 67.6, 67.3, 66.2, 63.3, 53.9, 50.1, 51.4, 48.1, 47.3, 42.6, 41.3, 40.0, 39.7–22.6 (aliphatic CH_2_) 13.4 (aliphatic CH_3_); [α]_D_^25^ = +48.0 (c 0.49, MeOH); EIMS: calcd for C_135_H_243_N_21_NaO_39_^+^ 2815.77 Found: 2816.45 [M+Na]^+^; Anal. Calcd for C_135_H_243_N_21_O_39_ C, 58.23; H, 8.80; N, 10.56; Found: C, 58.44; H, 9.10; N, 10.56.

*1,3,2',6',2''',6'''-Hexaammonium-guanidinyl-5''-deoxy-5''-N-(N,N-di-nonadecanoyl-Lys)-neomycin hexakis(trifluoroacetate)* (**4**). Yield = 84%; *R*_f_ 0.10 (NH_4_OH/MeOH/CH_2_Cl_2_ 2:9:4); ^1^H-NMR (300 MHz, CD_3_OD and CDCl_3_): δ 5.06 (m, 2H), 4.74 (m, 2H), 4.08 (m, 2H), 3.81 (m, 3H), 3.60 (m, 1H), 3.50 (m, 5H), 3.43 (m, 3H), 3.42–3.26 (m, 10H), 2.91 (m, 1H), 1.95 (dt, 4H, *J* = 6.3 Hz), 1.33 (m, 3H), 1.03 (s, 74H), 0.89 (t, 6H, *J* = 7.1 Hz); ^13^C-NMR (75 MHz, CD_3_OD): δ 177.2 (amide C=O), 176.4 (×2) (peptide amide C=O), 164.0–162.7 (TFA, q with *J ^2^_CF_* ~ 34.8 Hz), 159. 6 (×2), 159.4 (×3), 159.2 (guanidine C=NH), 124.1–112.4 (q with *J ^1^_CF_* ~ 292.0 Hz), 112.1, 99.2, 97.3 (anomeric carbons), 83.0, 79.2, 78.2, 75.9, 74.0, 73.8, 72.9, 72.4, 72.1, 71.9, 71.7, 70.8, 68.8, 61.9, 56.9, 55.1, 53.2, 51.8, 43.4, 43.0, 40.8, 40.0, 37.5–23.7 (aliphatic CH_3_) 14.7 (aliphatic CH_3_); [α]_D_^25^ = +42.0 (c 0.65, MeOH); EIMS: calcd for C_75_H_148_N_21_O_15_^+^ 1583.14 Found: 1583.14 [M+H]^+^; Anal. Calcd for C_87_H_153_F_18_N_21_O_27_ C, 46.09; H, 6.80; F, 15.08; N, 12.97; Found: C, 46.54; H, 7.22; F, 15.28; N, 13.19.

*5''-N-(Hexadecafluorodecanoyl)-1,3,2',6',2''',6'''-hexa-N-(tert-butoxycarbonyl-guanidino)-5''-deoxy-neomycin* (**6a**). Yield = 86%; R_f_ 0.36 (MeOH/CH_2_Cl_2_ 1:9); ^1^H-NMR (300 MHz, CD_3_OD): δ 5.88 (d, 1H, *J* = 3.68 Hz), 5.05 (dd, 2H, *J* =5.56, 1.70 Hz), 4.63–4.55 (m, 2H), 4.38 (m, 1H), 4.32–4.23 (m, 3H), 4.16 (m, 1H), 4.05–3.68 (m, 10H), 3.59–3.44 (m, 5H), 3.27 (m, 1H), 2.75–2.45 (m, 4H), 2.2 (m, 1H), 1.59–1.45 (m, 108H); ^13^C-NMR (75 MHz, CD_3_OD): δ 172.4,164.6.164.5, 164.3, 164.2, 164.1, 164.0, 158.8, 158.0, 157.9, 157.8, 157.6, 157.5, 154.7, 154.4, 154.2, 154.1, 154.0, 153.3, 153.2, 151.4, 140.5, 129.9, 127.7, 123.4, 120.4, 119.2, 114.9, 113.3, 99.3, 97.1, 89.1, 84.8, 84.7, 84.6, 84.5, 84.1, 84.0, 83.0, 80.7, 80.6, 80.5, 80.4, 80.3, 77.1, 76.7, 75.6, 74.3, 73.3, 72.0, 71.1, 68.1, 55.5, 53.0, 51.8, 50.2, 44.3, 44.0, 41.7, 35.2, 28.8–27.6 (Boc CH_3_ and aliphatic CH_2_).

*1,3,2',6',2''',6'''-Hexaammonium-guanidinyl-5''-deoxy-5''-N-(hexadecafluorodecanoyl-neomycin hexakis(trifluoroacetate)* (**6**). Yield = 91%; R_f_ 0.19 (NH_4_OH/MeOH/CH_2_Cl_2_ 1:5:5); ^1^H-NMRR (300 MHz, CD_3_OD): δ 5.91 (d, 1H, *J* = 2.9 Hz), 5.11 (d, 1H, *J* = 3.7 Hz), 5.01 (br s, 1H), 4.25–4.20 (m, 1H), 4.14 (dd, 1H, *J* = 6.2, 3.8 Hz), 4.08 (dd, 1H, *J* = 10.2, 6.0 Hz), 4.02 (dd, 1H, *J* = 5.6, 2.5 Hz), 3.98 (dd, 1H, *J* = 8.6, 4.2 Hz), 3.81 (m, 5H), 3.66–3.49 (m, 9H), 3.38 (m, 3H), 2.73–2.40 (m, 4H), 2.13 (m, 1H), 1.74 (m, 1H); ^13^C-NMR (75 MHz, CD_3_OD): δ 173.77 (NH-CO-), 163.3 (TFA), 159.62, 159.51, 159.30, 159.25, 159.18, 158.56 (–NHC(NH)NH2), 129.85, 123.85, 123.44, 120.45, 120.09, 119.21, 116.22 (F- attached carbons), 111.63, 100.14, 97.19 (anomeric carbons), 87.67, 82.18, 80.07, 77.50, 76.65, 75.60, 74.22, 74.04, 71.97, 71.50, 70.89, 68.81, 57.14, 55.36, 53.63, 51.75, 43.46, 43.14, 42.34, 33.79, 28.10, 27.57; EIMS: calcd. for C_40_H_63_F_17_N_19_O_13_+ 1340.45. Found: 1340.9 [M+H]^+^.

### 3.7. Determination of the MIC Values for Aminoglycoside-Lipid Conjugates ***1***–***6***

Bacterial isolates were obtained from the American Type Culture Collection (ATCC). Isolates were kept frozen in skim milk at −80 °C until minimum inhibitory concentration (MIC) testing was carried out. Following two subcultures from frozen stock, the *in vitro* activities of peptides were determined by macrobroth dilution in accordance with the Clinical and Laboratory Standards Institute (CLSI) 2006 guidelines [19]. Stock solutions of peptides were prepared and dilutions made as described by CLSI. Test tubes contained doubling antimicrobial dilutions of cation adjusted Mueller-Hinton broth and inoculated to achieve a final concentration of approximately 5 × 10^5^ CFU/mL then incubated in ambient air for 24 h prior to reading. Colony counts were performed periodically to confirm inocula. Quality control was performed using ATCC QC organisms.

### 3.8. Determination of the Hemolytic Activity for Aminoglycoside-Lipid Conjugates ***5***–***6***

Freshly isolated human erythrocytes isolated as recently described [20] were incubated for 1 h at 37 °C with 50, 100 µg/mL solution of peptide dissolved in PBS. The samples were centrifuged (4,000 rpm, 5 min) before the absorbance at 540 nm of the supernatant was measured by a microtiter negative and positive controls, respectively. Peptide concentrations corresponding to 50% hemolysis (EC50) were determined from the dose-response curves.

## 4. Conclusions

Six polycationic amphiphiles were synthesized, with the backbone of neomycin B providing cationic charge and either palmitic and arachidic bilipids or fluorinated undecanoic monolipid the hydrophobic region. The effect of basicity was evaluated by converting all six amine groups on neomycin B to guanidines, and all six compounds were tested against a panel of clinically relevant and ATCC Gram positive and Gram negative bacteria. Guanidinylation was found to increase activity 2–8 fold, with di-palmitoyl guandilyated neomycin B (**3**) displaying good activity against a number of Gram positive bacteria. When compared to previously synthesized monolipid PAs, compounds **1**–**6** displayed reduced activity. However, the PAs with fluorinated tails, **5** and **6**, had significantly less toxicity towards red blood cells, suggesting that fluorination may increase the therapeutic window of membrane-active amphiphiles.
